# Inferior vena cava thrombosis extension into the right atrium: An unusual case report of renal cell carcinoma

**DOI:** 10.1177/2050313X231204768

**Published:** 2023-10-06

**Authors:** Mara Trifoi, Marc Levine, Andrew Kim, Brandon Eden, Adam Albert

**Affiliations:** 1 Penn State College of Medicine, Hershey, PA, USA; 2 Lebanon VA Medical Center, Lebanon, PA, USA

**Keywords:** Inferior vena cava thrombosis, renal cell carcinoma

## Abstract

Inferior vena cava filling defects are common findings on computed tomography and magnetic resonance imaging, and accurate determination of pseudo, benign, or malignant thrombus is essential for clinical management. Inferior vena cava thrombosis involvement extending into the right atrium is a rare presentation of renal cell carcinoma. The degree of inferior vena cava and right atrium involvement is critical in determining management and prognosis of patients. Inferior vena cava thrombosis surgical thrombectomy is often a risky procedure due to the intraoperative determination of inferior vena cava thrombosis involvement. Accurate recognition of inferior vena cava thrombosis with right atrial involvement is critical in determining appropriate treatment options and preoperative level of involvement for surgical intervention. This case features a unique presentation of inferior vena cava thrombosis in renal cell carcinoma with right atrial involvement.

## Introduction

Inferior vena cava (IVC) filling defects are common findings on computed tomography (CT) and magnetic resonance imaging (MRI) images.^1^ The presence of pseudo-filling defects due to parallel renal vein vessels presents a diagnostic challenge. Accurate identification of an IVC filling defect as pseudo, benign, or malignant is essential for further clinical management.^1^ Renal cell carcinoma (RCC) is uniquely associated with a common complication of inferior vena cava thrombus (IVCT) formation, or malignant tumoral invasion of the IVC. Malignant thrombus of the IVC is a true misnomer in this setting as the more appropriate term is tumoral invasion of the vessel. RCC is associated with extension into the IVC in 4%–10% of cases. Venous tumor thrombus can even extend into the right atrium in 1%–3% of cases.^2^ The management of RCC with IVC involvement is common and helps to determine staging and subsequent treatment.^3^ We present a rare case of RCC with IVCT extension into the right atrium. Written consent for the following case report and images was attained through a legal representative of the deceased patient and are on file.

## Case report

A 79-year-old male with reduced ejection fraction heart failure, stage III chronic kidney disease, and diabetes mellitus was admitted for left foot cellulitis in the setting of worsening bilateral edema and acute renal failure (creatinine of 3.1 mg/dL with a baseline of 2.2 mg/dL).

**Figure 1.**
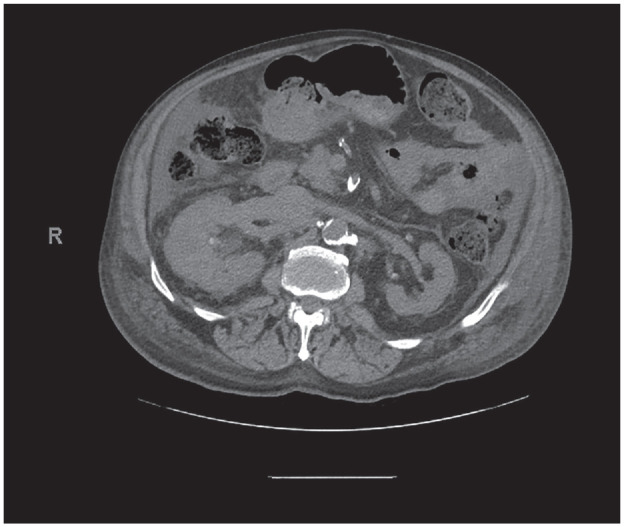
A noncontrast CT scan of the abdomen revealed asymmetric fullness of the right kidney with hydroureteronephrosis, with an increased density concerning for soft tissue neoplasm in the right ureter. There was a concern by the interpreting radiologist for an infiltrative renal mass (arrow).

**Figure 2.**
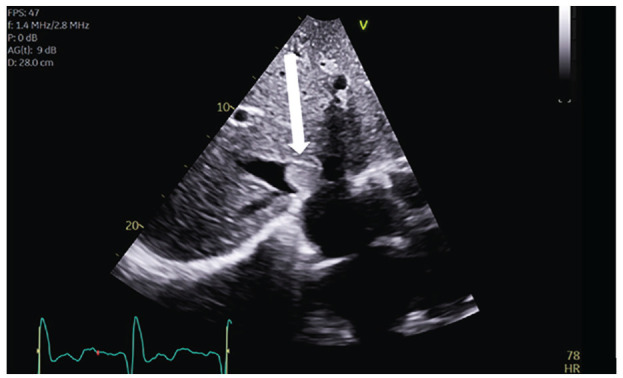
Transthoracic echocardiogram subcostal view revealed severe left ventricular dilation with an ejection fraction of 25%–30% and a mobile echodensity of 1.7 cm × 2.4 cm (arrow) at the junction of the inferior vena cava and the right atrium.

To evaluate the patient’s renal failure, a CT scan was performed which revealed right kidney enlargement with an infiltrative mass. In addition, the inferior vena cava was expanded and revealed an extensive thrombus (IVCT) (Figure 1). Echocardiogram revealed a mass at the junction between the IVC and right atrium (Figure 2). Subsequent MRI visualized the right renal vein mass extending into the left hepatic vein and IVC. The medulla and renal pelvis were thickened, and the collecting system was mildly dilated. The right renal vein was minimally enhanced, consistent with the presence of a thrombus. The patient was started on low-molecular-weight heparin, and further investigation was pursued.

A transjugular renal biopsy was performed to avoid seeding the abdomen. Pathology was conclusive for stage III (T3b, NO, MO) clear cell renal carcinoma. However, given his poor functional status and additional comorbidities, the patient elected hospice care.

## Discussion

IVC filling defects as identified on CT or MRI are common findings in patients with RCC, and the distinction between pseudo-filling defects and true underlying disease is critical. The nature of identified defects can be characterized as either pseudo, benign, or malignant in nature.^1^ CT images are often the initial imaging modality to identify an IVC filling defect with MRI images being utilized to help investigate unclear CT findings. Pseudothrombosis is the most frequent IVC filling defect observed on CT and can occur due to parallel laminar blood flow from the renal veins that can lead to an appearance resembling thrombosis.^4^ The use of delayed imaging techniques is typically adequate to confirm pseudothrombosis and exclude the presence of a true thrombus. Bland thrombus and malignant thrombus are differentiated by enhancement, contiguity of mass, F-FDG avidity, and vessel size.^4^ Transthoracic echocardiography can also be utilized in settings in which the right atrium is involved in an IVC thrombus.^2^ Bland thrombus is often associated with a hypercoagulable state due to causes such as oral contraceptive use, IVC filters, paraneoplastic syndromes, and vascular coagulopathies.^4^ Malignant tumor thrombus is associated with an expanded anterior-poster (AP) diameter of the renal vein ostium (RVo) and a complete level of occlusion of the IVC.^5^

The incidence of IVCT is rare and is often correlated with significant morbidity and mortality. The etiology of IVCT includes nephrolithiasis, RCC, pulmonary embolism, and liver disease.^6^ Categorization of IVCT as primary or secondary etiology is essential. Primary IVCT results from metabolic syndrome or hypertension processes, while secondary IVCT formation results from malignancy, surgery, or infectious causes.^2^ Assessment of hepatic and renal systems can elucidate the etiology of the thrombosis. Hematuria and flank pain suggest renal vein involvement, whereas ascites may be indicative of hepatic vein involvement.^7^

Management of RCC with malignant IVC thrombus depends on percent occlusion, acuity, and extent of proximal vascular involvement. Denotation of a thrombus as malignant or cruoric is critical prior to engaging in treatment considerations. Enhancement patterns on imaging are critical to determine the nature of thrombus. For a true bland thrombus, MRI does not show enhancement while a malignant thrombus shows mixed signals and enhancement on MRI.^4^ Standard initial treatment consists of anticoagulation, given the hypercoagulable state of the malignancy and the lack of a definitive diagnosis until further imaging and biopsy can be obtained. Catheter thrombolysis or thrombectomy may also be utilized in setting of an acute cruoric thrombus. In setting of a malignant thrombus, staging of RCC disease is highly dependent on the level of involvement. Surgical options are dependent on involvement and include IVC filter placement, stapling, and ligating the IVC inferior to the malignant thrombus, and segmental resection of the IVC.^8^ Prognosis for patients who do not undergo caval thrombectomy and resection for RCC and IVCT is 5 months, while those who undergo surgical treatment have a 40%–60% 5-year survival rate.^9^ Surgical considerations are dependent on the level of IVC involvement and often high risk due to the fact that true level of IVC involvement is often only able to be determined intraoperatively. AP diameter at the renal vein opening from MRI or CT scans can help to determine IVC thrombus involvement preoperatively.^5^ This highlights the importance of accurate recognition of the IVCT level of extension in diagnostic imaging modalities.

## Conclusion

IVC filling defects are common findings on CT and MRI, and accurate determination of pseudo, benign, or malignant thrombus is essential for clinical management. IVCT involvement extending into the right atrium is a rare presentation of RCC. The degree of IVC and right atrium involvement is critical in determining management and prognosis of patients. IVCT surgical thrombectomy is often a risky procedure due to intraoperative determination of IVCT involvement. CT and MRI images can be utilized to determine the AP diameter of the renal vein opening which can help determine the preoperative level of involvement. This case features a unique presentation of IVCT in RCC with right atrial involvement that can help future clinicians better identify such involvement and its implications in regards to management.
